# The Plasma Oxylipidome Links Smoking Status to Peripheral Artery Disease

**DOI:** 10.3390/metabo12070627

**Published:** 2022-07-07

**Authors:** Stephanie P. B. Caligiuri, Grant N. Pierce, Amir Ravandi, Harold M. Aukema

**Affiliations:** 1 Nash Family Department of Neuroscience, Icahn School of Medicine at Mount Sinai, New York, NY 10029, USA; 2 Canadian Centre for Agri-Food Research in Health and Medicine, Albrechtsen Research Centre, St. Boniface Hospital, Winnipeg, MB R2H 2A6, Canada; gpierce@sbrc.ca (G.N.P.); harold.aukema@umanitoba.ca (H.M.A.); 3 Institute of Cardiovascular Sciences, Albrechtsen Research Centre, St. Boniface Hospital, Winnipeg, MB R2H 2A6, Canada; aravandi@sbrc.ca; 4 Department of Physiology and Pathophysiology, University of Manitoba, Winnipeg, MB R3E 0J9, Canada; 5 Department of Internal Medicine, University of Manitoba, Winnipeg, MB R3E 0Z2, Canada; 6 Department of Food and Human Nutritional Sciences, University of Manitoba, Winnipeg, MB R3T 6C5, Canada

**Keywords:** eicosanoids, lipidome, peripheral artery disease, tobacco, smoking, oxylipins

## Abstract

Peripheral artery disease (PAD) is prevalent among individuals with a history of tobacco smoking. Although oxidation of lipids may contribute to atherogenesis in vascular disease, enzymatically and nonenzymatically produced oxidized lipids can have varying and contrasting physiological effects. The underlying mechanisms of atherogenic vulnerability can be better elucidated with the recent advances in oxylipidome quantification using HPLC-MS/MS technology. In a randomized, controlled clinical trial, the plasma oxylipidome was analyzed in participants living with PAD by smoking status (n = 98) and in nonsmoking comparators without chronic disease (n = 20). Individuals with PAD had approximately a four-fold higher level of total plasma oxylipins versus the comparator. Cessation of smoking in individuals with PAD was associated with significantly lower levels of linoleic acid-derived TriHOMEs, greater levels of omega-3 fatty acid-derived oxylipins, and greater levels of nonfragmented oxidized phosphatidylcholines (OxPCs). Individuals living with PAD but without a history of smoking, exhibited higher levels of the putative atherogenic fragmented OxPCs versus individuals who currently or previously smoked. These data implicate the plasma oxylipidome in PAD and that smoking cessation is associated with a less inflammatory profile. Furthermore, fragmented OxPCs may play a more significant role in the pathophysiology of PAD in individuals without a history of smoking.

## 1. Summary

The etiology of peripheral artery disease (PAD) has been ascribed to a multitude of factors, of which tobacco use is significant [1]. Smoking of tobacco products is thought to induce oxidative stress by influencing endothelial function and oxidation of lipid molecules. These changes in oxidation are suggested to be mechanisms by which smoking may lead to the development of atherosclerosis [2]. However, in the last decade, the ability to quantify specific oxidized lipid species has led to an appreciation that oxidized lipids can have contrasting effects [3,4]. For example, some oxylipins may display vasodilatory or inflammation resolving functions, while others may propagate vasoconstriction and inflammation [3,4]. To further add complexity, the same oxylipin may exhibit contrasting effects depending on the receptor to which it binds [5]. Because of the significant role oxidized lipids appear to have in inflammation, vascular tone, and coagulation, it is clear that the oxylipidome can be a significant therapeutic target. Oxylipins, which include the octadecanoids, eicosanoids, and docosanoids, have been targeted for decades with one of the most highly utilized class of drugs, nonsteroidal antiinflammatory drugs, to reduce thrombosis, inflammation, and pain [6]. The ability to more elegantly target specific oxidized lipids for therapy can be enhanced with an understanding of how the concentrations of specific oxidized lipid species may change in different disease states and lifestyle factors.

The oxylipidome, a class of both enzymatically and nonenzymatically oxidized lipids, including oxylipins and oxidized phosphatidylcholines (OxPCs), has been significantly implicated in inflammation, vascular tone, atherogenesis, and vascular disease [3,7]. Recent advances in lipidomics quantification using high-performance liquid chromatography-mass spectrometry coupling (HPLC-MS/MS) have led to a greater understanding of how these bioactive molecules may change in disease states and the role they may have in the amelioration or progression of disease. For example, specific plasma oxylipins, such as the DiHETrEs, thromboxane B_2_, and fragmented OxPCs, were associated with acute coronary events in patients with PAD [7,8]. A multitude of plasma oxylipins, differed in younger versus older individuals [9]. Older individuals tended to have higher concentrations of many docosahexaenoic acid (DHA)- and arachidonic acid (ARA)-derived oxylipins versus their younger counterparts. As oxylipins are produced from lipids, great interest exists around dietary interventions focusing on lipid intake as therapeutic strategies. Consumption of flaxseed, a dietary source of the omega-3 polyunsaturated fatty acid, alpha-linolenic acid (ALA), reduced the disparity in the plasma oxylipin profile between the younger and older individuals [9]. Similarly, consumption of dietary flaxseed reduced the concentration of soluble epoxide hydrolase-derived oxylipins known to influence vascular constriction; this was associated with a reduction in blood pressure in patients living with PAD [10,11]. Given the extent to which tobacco use contributes to PAD [1], and the influence of tobacco smoking on the inflammatory oxidative state [12], it is important to assess the role of tobacco smoking status on the plasma oxylipidome. The aim of the current study was to characterize the plasma oxylipidome profile, which included oxylipins and OxPCs, of participants living with PAD by their tobacco smoking status.

## 2. Results

### 2.1. Participant Characterstics

Participant details, such as plasma lipid and plasma oxylipin profiles of the comparator group (n = 20) living without chronic disease and not smoking, have been previously published [9,13,14]. Of the 98 participants living with PAD, the majority (66%) were previous tobacco smokers with an average of 13 years since last smoking. Individuals without a history of smoking comprised 7% of the study population, and currently smoking individuals comprised 27%. The participants in their respective tobacco exposure groups did not significantly differ by gender, body mass index (BMI), cholesterol levels, or most plasma polyunsaturated fatty acid levels. However, the groups did differ by age and their concentration of plasma long-chain omega-3 fatty acids, eicosapentanoic acid (EPA), and docosahexaenoic acid (DHA) (*p*-value < 0.05) (Table 1). Thus, consideration of age and plasma omega-3 fatty acids in the context of the oxylipidome profile was included in regression modelling to control for such factors.

### 2.2. Plasma Oxylipin Profile

**Figure 1:**
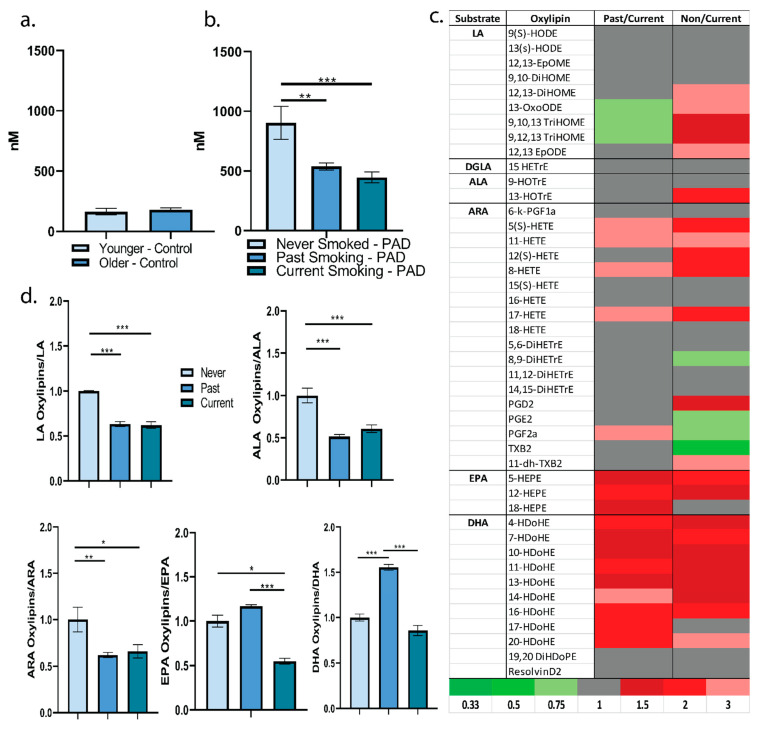
(**a**) Total plasma oxylipin concentration (nM) in nonsmoking individuals aged 19–28 years (younger) and 45–64 years (older). (**b**) Total plasma oxylipin concentration (nM) by smoking status in patients living with PAD. One way ANOVA (F = 9.334, *p*-value = 0.0002). Tukey’s post-hoc: **—Never Smoked vs. Past (*p* = 0.0011), ***—Never Smoked vs. Current (*p* = 0.0001). (**c**) Heat map representing fold differences in plasma concentrations of oxylipins by smoking status. (**d**) Ratio of plasma oxylipins to plasma fatty acid substrate relative to the Never Smoked group. LA: ANOVA ***—(F = 9.92, *p*-value = 0.0001). Tukey’s post-hoc: ***—Never Smoked vs. Past (*p* = 0.0001), ***—Never Smoked vs. Current (*p* = 0.0002). ALA: ANOVA (F = 18.3, *p*-value < 0.0001). Tukey’s post-hoc: ***—Never Smoked vs. Past (*p* < 0.0001), ***—Never Smoked vs. Current (*p* < 0.0001). ARA: ANOVA (F = 5.92, *p*-value = 0.0038). Tukey’s post-hoc: **—Never Smoked vs. Past (*p* = 0.0025), *—Never Smoked vs. Current (*p* = 0.014). EPA: ANOVA (F = 27.9, *p*-value < 0.0001). Tukey’s post-hoc: ***—Past Smoked vs. Current (*p* < 0.0001), *—Never Smoked vs. Current (*p* = 0.0108). DHA: ANOVA (F = 70.9, *p*-value < 0.0001). Tukey’s post-hoc: ***—Never Smoked vs. Past (*p* < 0.0001), ***—Past Smoked vs. Current (*p* < 0.0001).

Consideration of total plasma oxylipin concentration can provide insight into the extent of enzymatic PUFA oxidation. Individuals in the comparator group, without chronic disease and not currently smoking, exhibited approximately 150 nM in total level of plasma oxylipins; age did not appear to influence total level of plasma oxylipins in this population (Figure 1a). Individuals living with PAD exhibited approximately a four-fold higher level of total plasma oxylipins versus individuals without chronic disease (Figure 1b). Thus, PAD may be associated with a greater production, less degradation/elimination, or increased translocation to plasma of oxylipins. In regard to smoking status in individuals living with PAD, individuals who never smoked tobacco products had a significantly higher level of total circulating plasma oxylipins versus those with a past history of smoking or currently smoking. There was no significant difference for total plasma oxylipin level between those who currently smoked and those who quit smoking. Thus, individuals living with PAD with no tobacco smoking history may enzymatically produce more oxylipins from polyunsaturated fatty acids, exhibit less degradation/elimination of oxylipins, or exhibit a greater release of oxylipins to the plasma (Figure 1b)**.** More specifically, of the 45 plasma oxylipins detected, 24 oxylipins were at least 1.5-fold higher in individuals who never smoked versus individuals currently smoking. Primarily, these included the omega-3 fatty acid-derived oxylipins. In addition, some omega-6 fatty acid-derived oxylipins with the potential to propagate inflammation and coagulation, such as thromboxane B_2_ and prostaglandins F_2__α,_ E_2_, were lower in individuals who never smoked. By comparison, 18 oxylipins displayed at least 1.5-fold higher levels between those who quit smoking versus currently smoking. These were primarily omega-3-derived oxylipins (Figure 1c). Absolute concentrations of the different plasma oxylipins by smoking status are in Supplemental Table S1. Because of the difference in plasma EPA and DHA among the smoking categories, normalization to plasma polyunsaturated fatty acids was important to consider. When plasma oxylipins were normalized to their substrate content in the plasma, individuals living with PAD that never smoked appeared to have more oxylipins derived from linoleic acid, alpha-linolenic acid, and arachidonic acid. However, this pattern did not continue into the long-chain omega-3 fatty acids (Figure 1c). Individuals with PAD that quit smoking appeared to have greater EPA and DHA-derived oxylipins versus individuals who currently or never smoked (*p* < 0.05) (Figure 1d). This suggests either less degradation of these oxylipins or an upregulation of putative anti-inflammatory oxylipins with the cessation of smoking in PAD. Next, we controlled for the differing variables in this population with regression modeling: age, plasma fatty acid concentration, and smoking status. With these factors included in the regression model, the oxylipins most influenced by their substrate were the linoleic acid-derived oxylipins. More specifically, out of all 45 oxylipins detected, 6 oxylipins were significantly and positively associated to their level of substrate in the plasma, all of which were linoleic acid-derived: 12,13-EpODE, 12,13-EpOME, 13-HODE, 13-OXoODE, 9-HODE, and 9,10-DiHOME. For example, for every 1 mg/mL increase in plasma linoleic acid, there is a predicted increase of 102 nM of 12,13-EpODE (Table 2). This may reflect the significant potential of dietary LA intake to influence these specific oxylipins in this population. Number of years since smoking cessation, duration of smoking, and number of cigarettes smoked per day were not significantly associated to level of individual plasma oxylipins.

### 2.3. Oxidized Phosphatidylcholines

**Figure 2:**
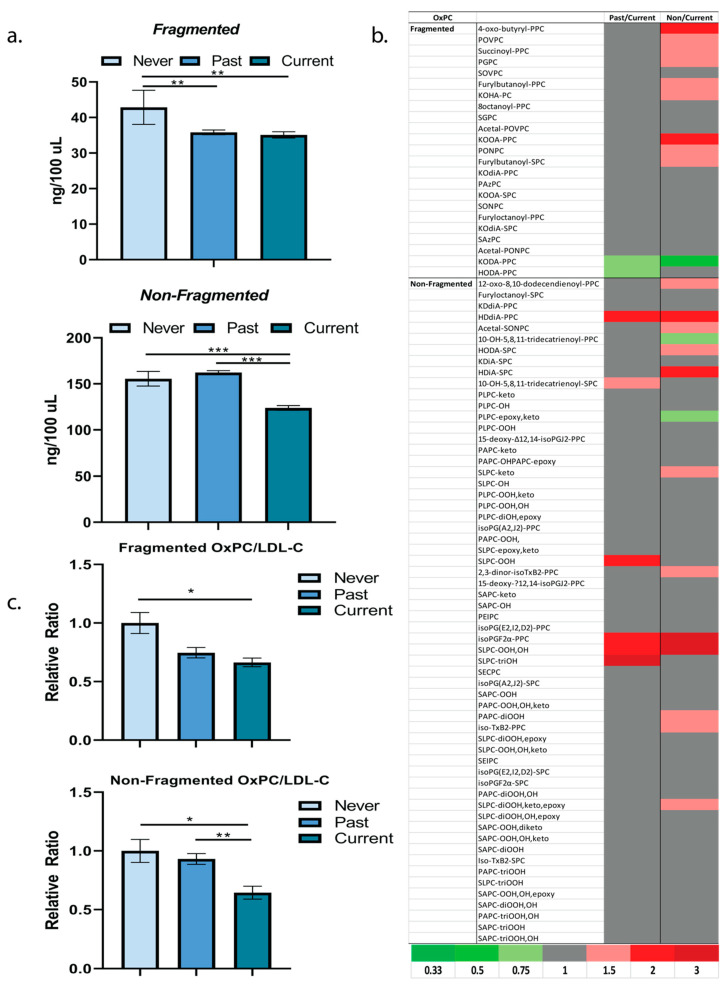
(**a**) Total plasma fragmented and nonfragmented OxPC concentration by smoking status in patients living with PAD. Fragmented: One way ANOVA (F = 5.19, *p*-value = 0.0073). Tukey’s post—hoc: **—Never Smoked vs. Past (*p* = 0.0086), **—Never Smoked vs. Current (*p* = 0.0064). Nonfragmented: One way ANOVA (F = 56.21, *p*-value < 0.0001). Tukey’s post-hoc: ***—Past Smoked vs. Current (*p* < 0.0001), ***—Never Smoked vs. Current (*p* < 0.0001). (**b**) Heat map representing fold differences in plasma concentrations of OxPCs by smoking status. (**c**) Plasma fragmented and nonfragmented OxPC concentrations relative to LDL-cholesterol levels by smoking status in patients living with PAD. Fragmented/LDL-C: One-Way ANOVA: (F = 3.20, *p*-value = 0.045). Tukey’s post-hoc: *—Never Smoked vs. Current (*p* = 0.035). Nonfragmented/LDL-C: One-Way ANOVA: (F = 7.16, *p*-value = 0.0013). Tukey’s post-hoc: *—Never Smoked vs. Current (*p* = 0.043), **—Past Smoked vs. Current (*p* = 0.0014).

OxPCs are divided into fragmented and nonfragmented molecules (Figure 2a); the fragmented molecules represent a class of nonenzymatically oxidized lipids that are associated with acute coronary events, ischemia, and with significant atherogenic and inflammatory potential [7,15,16]. In this participant population living with PAD, individuals with no history of tobacco use had significantly higher total concentrations of both fragmented and nonfragmented OxPCs versus individuals with a history of tobacco use (Figure 2a). Nine fragmented OxPCs were at least 1.5-fold higher in individuals living with PAD that never smoked versus currently smoking individuals (Figure 2b). This may reflect a significant influence of OxPCs in the pathophysiology of PAD in the absence of tobacco use. In line with the potential improvement in the plasma oxylipin profile with cessation of smoking, 2 fragmented OxPCs, KODA-PPC and HODA-PPC were at least 0.75-fold lower versus currently smoking individuals (Figure 2b). By comparison to plasma oxylipins, the proportion of individual OxPCs that differed by smoking status was less. For example, of the 83 plasma OxPCs detected, 6 OxPCs were at least 1.5-fold higher in individuals with past versus current smoking. By comparison, 21 of the OxPCs were at least 1.5-fold higher among individuals who never smoked and individuals currently smoking (Figure 2b). Absolute concentrations of plasma OxPCs by smoking status are in Supplemental Table S2. Despite blood cholesterol levels not differing by smoking status in this population, we normalized plasma OxPC content to LDL-C, a significant source of lipids for OxPC production; the same pattern in differing plasma OxPC levels by smoking status were noted as without normalization (Figure 2c). When controlling for lifestyle factors, such as age, gender, and BMI, smoking status was a significant predictor of only one fragmented OxPC, KODA-PPC (regression estimate: 1.09 ± 0.33, *p* = 0.0013). Plasma KODA-PPC was significantly elevated in the currently smoking group versus never or past smoking individuals. In currently smoking individuals, duration of smoking (−0.0115 ± 0.00376, *p* = 0.0062) and number of cigarettes per day (0.0128 ± 0.00531, *p* = 0.0247) significantly predicted the plasma level of SLPC-diOOH, epoxy when age, gender, and BMI were controlled. These specific OxPCs represent potential targets that are sensitive to tobacco exposure.

## 3. Discussion

Major breakthroughs in our understanding of atherogenesis and vascular disease have come from the latest advances in expansive lipidomics analyses. For example, in the last decade, it has been elucidated that hundreds of different oxidized lipids can have expansive and even contrasting effects on inflammation, immunity, vascular tone, and coagulation [3,4,8,15,16]. This understanding has led to the targeting of specific enzymes, substrates, or oxylipins for therapeutic purposes. For example, enhancing dietary sources of different omega-3 fatty acids may give rise to their oxylipin products, which appear to influence levels of inflammation, vascular tone, and disease progression in humans and animal models of cardiovascular and renal disease [9,10,11,17]. Targeting the production of specific inflammation resolving oxylipins, such as the maresins and resolvins, is of great interest in acute and chronic inflammation [18,19]. Targeting of enzymes, such as soluble epoxide hydrolase to reduce the production of vascular tone regulating oxylipins, DiHETrEs, may result in blood pressure reduction [11]. Notably, soluble epoxide hydrolase has been a target of interest to reduce nicotine and tobacco-induced inflammation and vasoconstriction [20,21]. However, a lower level in only one soluble epoxide hydrolase product, 8-9-DiHETrE, was observed in the present study in individuals who never smoked versus individuals currently smoking.

Understanding how to ameliorate the impact of tobacco exposure is still of great need, as in the last several years tobacco product use has risen. In 2021, it was estimated that 34% of high school students frequently used tobacco products, such as e-cigarettes, cigarettes, cigars, hookah, or nicotine pouches [22]. This is a rise in tobacco product use from previous years. It is generally recognized that tobacco exposure may induce oxidative stress, inflammation, and vascular constriction [12]. The vehicle, flavorants, and nicotine itself in vapes and e-cigarettes, albeit to a lesser extent, still appear to induce oxidative stress and/or apoptosis [23,24,25]. Nicotine itself may have negative effects on cell signaling and risk for chronic disease, such as diabetes [26]. However, only recently, we have gained a better understanding of how smoking tobacco products or the cessation of smoking may influence specific lipid molecules to exacerbate or ameliorate the subsequent pathophysiological sequelae.

### 3.1. Linking the Plasma Oxylipidome to PAD

The results of the present study add to the understanding of how smoking status may influence the plasma oxylipidome in patients living with PAD. The current results suggest PAD itself may be associated with an upregulated production or downregulated degradation of plasma oxylipins versus individuals without chronic disease. Cessation of smoking was associated with differences in the plasma oxylipidome versus individuals currently smoking. These differences included higher levels of omega-3 fatty acid-derived oxylipins and lower levels of LA-derived oxylipins. Based on our current understanding of these specific oxylipins, these differences suggest an anti-atherogenic effect upon cessation of smoking. As such, this data supports the cessation of smoking in PAD and suggests a potential lipid-mediated protection against atherogenesis. Downregulation of other pro-atherogenic molecules upon smoking cessation, such as microRNA 27b, has been observed in participants living with PAD [27]. Beyond smoking cessation, diet may influence the oxylipidome as well. In this population, the LA-derived oxylipins correlated significantly to substrate content, plasma LA. This suggests that it is possible to influence oxylipin concentrations by controlling dietary sources of LA in a population with PAD. It is well recognized that dietary sources of PUFAs can significantly alter the plasma fatty acid and oxylipin profile. For example, intake of 6 g of the omega-3 fatty acid alpha-linolenic acid via ground flaxseed was able to significantly increase plasma ALA in this and other populations [13,28]. In contrast, lowering the dietary intake of the omega-6 fatty acid linoleic acid has resulted in reduced plasma LA and reduced concentrations of oxylipins derived from LA [29]. Thus, dietary intervention may prove to be an effective strategy by which to target the oxylipidome in individuals living with PAD.

A surprising finding of this study included the elevated level of total oxylipins and pro-atherogenic fragmented OxPCs in individuals living with PAD but with no smoking history. This was observed despite a lower level of some arachidonic acid-derived oxylipins with atherogenic potential (i.e., 8,9-DiHETrE, thromboxane B_2_, and prostaglandins F_2_/E_2_). This suggests a contrasting effect between the oxylipins and OxPCs in individuals with PAD and no smoking history. As tobacco use is a significant contributing factor to PAD [1], it begs the question how PAD may develop in individuals without tobacco exposure. These results suggest a greater importance of fragmented OxPCs in the onset and progression of PAD in individuals without tobacco exposure versus those with tobacco use. Many factors beyond tobacco exposure may influence the production of oxidized lipid species, such as PUFA intake [3,9,17], exposure to air pollution [30], and high intake of fried foods [31] to name a few. Thus, individuals living with PAD may have a particularly elevated level of proatherogenic OxPCs due to reasons other than tobacco exposure that may underlie the pathophysiological mechanism.

### 3.2. Limitations

A limitation of this study includes the small sample size of individuals with no smoking history; however, this reflects the realistic distribution of cigarette smoking status in individuals living with PAD. The pathophysiology of PAD is linked to cigarette smoking and this small subpopulation provides insight into the etiology in the absence of a smoking history.

### 3.3. Conclusions

In conclusion, the current study reports that smoking status in patients with PAD is associated with differences in the plasma oxylipidome. This adds to the understanding of how various oxidized lipid species may play a role in PAD in the absence and presence of tobacco use. For example, fragmented OxPCs may explain a greater extent of the pathophysiology of PAD in individuals with no history of tobacco use. The cessation of smoking was associated with a greater concentration of long-chain omega-3-derived oxylipins and a lower concentration of LA-derived oxylipins. These data support a potential benefit of smoking cessation in PAD due to alterations in the oxylipidome. Using regression modeling, LA-derived oxylipins were significantly associated to plasma LA levels. As such, these data imply that dietary interventions targeting LA may influence the plasma oxylipidome in patients with PAD and may provide therapeutic targets.

## 4. Methods

### 4.1. Clinical Trials

Study participant details have been previously published [14,28,32]. The clinical trial registry for the Flax-PAD trial is available at http://www.clinicaltrials.gov/ct2/show/NCT00781950?term=grant+pierce+flax&rank=1 (accessed on 1 June 2022). All participants provided informed consent, and all procedures were performed according to institutional guidelines. The Age Dependency study included 20 individuals with no clinical indications of chronic disease and not currently smoking. The individuals were grouped into younger (19–28 years) and older categories (45–64 years). This group served as the comparator for patients living with PAD. The Flax-PAD trial was approved by Health Canada and its Natural Health Product Directorate, the University of Manitoba Research Ethics Board, and the St Boniface Hospital Research Review Committee. Briefly, 110 participants diagnosed with peripheral artery disease were recruited to determine the impact of 30 g of ground flaxseed or control food products on parameters of cardiovascular health over a period of one year. The data herein include analyses of blood samples acquired at baseline. In both cohorts, blood was collected from the median cubital vein into EDTA tubes, spun down at 1200 g for 10 min, plasma was drawn and then stored at −80 °C until oxylipin and OxPC analysis.

### 4.2. Oxylipidome

A total of 98 participants living with PAD and 20 participants living without chronic disease had adequate plasma (200 µL) for oxylipidomic analyses. The oxylipin and OxPC extraction and analysis methodology have been previously published [7,17]. Briefly, 200 uL of plasma was acidified to pH 3 with HCl and deuterated internal standards added as previously published. Strata-X, 33 μm, polymeric reversed phase columns (Phenomenex, product no. 8B-S100-UBL) were conditioned with 2 mL of methanol (LC-MS grade), followed by 2 mL of pH 3 LC-MS grade water. The sample was applied to the column, followed by a 10% methanol in pH 3 water wash that was rinsed from the tube previously holding the sample. To elute, 2 mL of methanol was added to the column. Then, this eluate was dried under a nitrogen water bath set to 37 °C and reconstituted with 100 μL of solvent A (water:acetonitrile:formic acid (70:30:0.02 *v*/*v*/*v* LC-MS grade). A Prominence HPLC system (Shimadzu Corporation) was coupled to a 4000 QTRAP triple quadrupole linear ion trap hybrid mass spectrometer equipped with a Turbo V electrospray ion source (AB Sciex, Framingham, Massachusetts, USA). The same sample eluate from solid phase extraction was run for oxylipins and OxPCs. A Luna 5 µm C18 column (Phenomenex product number: 00G-4252-B0, 250 × 2.0 mm) was used for oxylipin separation and a Ascentis Express C18 HPLC column (15 cm × 2.1 mm, 2.7 μm; Supelco Analytical) was used for OxPC separation. Analytes were detected with multiple reaction monitoring. Chromatograms were manually analyzed using Analyst Version 1.5 (Applied Biosystems Concord, ON). The quantification limit was set to a standard of 3 levels above background. Quantification of oxylipins and OxPCs was determined using the stable isotope dilution method and represents the free pool as nM for oxylipins and ng/100 uL for oxPCs [33]. To allow for across cohort comparison, absolute quantitation was utilized, a pooled quality control sample was run before and intermittently between samples, internal deuterated standards were added to every participant sample, and the same equipment and methodology were utilized. Details of the deuterated internal standards, collision-induced dissociation mass transitions, retention times, and detector response factors for analytes were previously published [17]. Plasma fatty data from participants and methods were previously published by control and intervention flaxseed groupings [14,28].

### 4.3. Statistical Analyses

Statistical analyses were performed using Statistical Analysis Software (SAS, version 9.3) and GraphPad Prism Software (version 9.3). Oxylipins and OxPC data were treated as continuous variables and assessed for Gaussian distribution using Shapiro–Wilk’s. ANOVA were run on continuous variables to assess for differences by smoking status. Tukey’s was utilized for post-hoc comparisons. Regression modeling was performed to determine any significant association of smoking status, age, gender, BMI, and fatty acid levels on oxylipin concentrations. All statistical analyses were set to a significance level of 0.05.

## Figures and Tables

**Table 1:** Participant Characteristics from the Flax-PAD trial by Smoking Status.

	Never Smoked (n = 7) Average ± Standard Deviation	Quit Smoking (n = 65) Average ± Standard Deviation	Currently Smoking (n = 26) Average ± Standard Deviation	*p*-Value
Age (years)	71 ± 6.6	68 ± 8.8	63 ± 7.7 *	0.0091
Gender (M/F)	5/2	48/17	19/7	0.29
Body Mass Index	25 ± 4.7	28 ± 4.4	28 ± 5.0	0.29
Duration of Smoking (years)	N/A	31 ± 12	37 ± 8.2 **	0.044
Number of Cigarettes/Day	N/A	24 ± 9.8	15 ± 9.5 ***	0.00030
Time Since Quit Smoking (years)	N/A	13 ± 13	N/A	N/A
Total Cholesterol (mmol/L)	4.2 ± 1.1	4.4 ± 1.1	4.5 ± 1.2	0.81
LDL-C (mmol/L)	2.1 ± 0.90	2.4 ± 0.92	2.6 ± 1.0	0.32
HDL-C (mmol/L)	1.3 ± 0.30	1.2 ± 0.33	1.1 ± 0.17	0.19
Linoleic Acid (mg/mL)	0.64 ± 0.21	0.71 ± 0.19	0.67 ± 0.21	0.50
Alpha-Linolenic Acid (mg/mL)	0.019 ± 0.011	0.020 ± 0.012	0.017 ± 0.011	0.61
Arachidonic Acid (mg/mL)	0.21 ± 0.034	0.21 ± 0.066	0.20 ± 0.046	0.44
Eicosapentanoic Acid (mg/mL)	0.030 ± 0.013	0.021 ± 0.010	0.017 ± 0.0090 ****	0.0099
Docosahexaenoic Acid (mg/mL)	0.055 ± 0.017	0.041 ± 0.013 *****	0.035 ± 0.014 *****	0.0031

One-Way ANOVA, post-hoc Tukey’s: * quit smoking vs. current smoking *p*-value 0.0139; ** never smoked vs. quit smoking *p*-value < 0.0001; *** quit smoking vs. currently smoking *p*-value = 0.0051; **** never vs. current smoking for EPA *p*-value = 0.0080; ***** never vs. current smoking for DHA (*p*-value = 0.0023); and never vs. past smoking (*p*-value = 0.030).

**Table 2:** Statistically Significant Regression Models Predicting Plasma Oxylipin Concentrations in Patients Living with PAD [Estimate (Standard Error of the Mean)].

Oxylipin	Intercept	Age	Smoking Status	Fatty Acid Substrate (mg/mL)	R-Squared
12,13-EpODE	−96.0 (63.9)	1.78 (0.721)	−4.84 (11.5)	102 (31.0) **	0.176
12,13-EpOME	0.863 (27.3)	−0.0319 (0.309)	−0.175 (4.91)	54.5 (13.3) ***	0.158
13-HODE	2.08 (8.37)	−0.0314 (0.0944)	−0.653 (1.50)	16.7 (4.06) ***	0.161
13-OxoODE	0.865 (20.3)	−0.159 (0.229)	−0.0505 (3.64)	27.1 (9.84) **	0.0809
9-HODE	−1.03 (7.97)	0.0184 (0.0900)	−0.425 (1.43)	13.77 (3.87) ***	0.127
9,10-DiHOME	1.47 (3.75)	−0.0500 (0.0424)	−0.537 (0.672)	9.71 (1.82) ***	0.249

**—Indicates 0.0001 < *p*-value ≤ 0.0099, ***—Indicates *p*-value < 0.0001.

## Data Availability

Data is contained within the article or supplementary material. The data presented in this study are available in Supplemental Tables S1 and S2.

## Supplementary Materials

The following supporting information can be downloaded at: https://www.mdpi.com/article/10.3390/metabo12070627/s1, Table S1: Individual Plasma Oxylipins by Smoking Status (nM) Mean and Standard Deviation; Table S2: Individual Plasma OxPCs by Smoking Status (ng/100µL) Mean and Standard Deviation.

## Abbreviations

ALA: alpha-linolenic acid, ARA: arachidonic acid, DGLA: dihomo-γ-linolenic acid, DiHDPA: dihydroxydocosapentanoic acid, DIHETRE: dihydroxyeicosatrienoic acid, DiHOME: dihydroxyoctadecenoic acid, DHA: docosahexanoic acid, EPA: eicosapentanoic acid, EpOME: epoxyoctadecenoic acid, HDoHE: hydroxydocosahexanoic acid, HEPE: hydroxyeicosapentanoic acid, HETE: hydroxyeicosatetraenoic acid, HETrE: hydroxyeicosatrienoic acid, HHTrE: hydroxyheptadecatrienoic acid, HODE: hydroxyoctadecadienoic acid, HOTrE: hydroxyoctadecatrienoic acid, LA: linoleic acid, oxoODE: oxooctadecadienoic acid, OXoOTrE: oxooctadecatrienoic acid, OxPC: oxidized phosphatidylcholine, PAD: peripheral artery disease, PUFA: polyunsaturated fatty acids, TriHOME: trihydroxyoctadecenoic acid, POVPC: 1-palmitoyl-2-(5′-oxo-valeroyl)-sn-glycero-3-phosphocholine, PGPC: 1-palmitoyl- 2-glutaryl-sn-glycero-3-phosphocholine, SOVPC: sn-1-stearoyl-2-oxovaleroyl-PC, KOHA-PC: 1-palmityl-2-(4-oxo- 7-oxohept-5-enoyl)-sn-glycero-3-phosphoserine, SGPC: 1-stearoyl-2-glutaroyl-sn-glycero-3-phosphocholine, KOOA-PC: 5-hydroxy-8-oxo-6-octenoic acid ester of 2-lysophosphatidylcholine, PONPC: 1-palmitoyl-2-(9′-oxo-nonanoyl)-sn-glycero-3-phosphocholine, KOdiA-PC: 1-palmitoyl-2-(5′-keto-6′-octenedioyl)-sn-glycero- 3-phosphocholine, PAzPC: 1-palmityl-2-azelayl-sn-glycero-3-phosphocholine, KOOA-SPC, SONPC: 1-stearoyl-2-(9-oxo-nonanoyl)-snglycero-3-phosphocholine, SAzPC: 1-stearoyl-2-azelaoyl-sn-glycero-3-phosphocholine, HODA-PC: 9-hydroxy-12-oxododec-10-enoic acid ester of 2-lysophosphatidylcholine, HDdiA-PC: 1-palmitoyl-2-(9-hydroxy-11-carboxyundec-6-enoyl)-sn-glycero3-phosphocholine. PLPC: 1-palmitoyl-2-linoleoyl-sn-glycero-3-phosphocholine, PAPC: 1-palmitoyl-2-arachidonoyl-snglycero-3-phosphocholine, SLPC: 1-stearoyl-2-linoleoyl-sn-glycero-3-phosphocholine, PGJ2-SPC: prostaglandin J2-stearoyl phosphocholine, SAPC: 1-stearoyl-2-arachidonoyl-sn-glycero-3-phosphocholine, PEIPC: 1-palmitoyl-2-(5,6-epoxyisoprostane E2)-sn-glycero-3-phosphocholine, SEIPC: 1-stearoyl-2-(5,6-epoxyisoprostane E2)-sn-glycero-3-phosphocholine, OH: hydroxyl, OOH: hydroperoxy.
