# Supplementary material for: The Plasma Oxylipidome Links Smoking Status to Peripheral Artery Disease
Source: Metabolites. 2022 Jul 7;12(7):627. doi: 10.3390/metabo12070627 (PMC9317423; doi:10.3390/metabo12070627)
Supplement: Supplementary file 1 [file metabolites-12-00627-s001.zip › metabolites-1794964-supplementary.pdf]

Supplemental Table S1 – Individual Plasma Oxylipins by Smoking Status (nM) Mean and Standard Deviation

Oxylipin	Never Smoked		Quit Smoking		Currently Smoking	
	**Mean**	**SD**	**Mean**	**SD**	**Mean**	**SD**
**10-HDoHE**	0.924	1.145	0.590	1.217	0.165	0.205
**11,12-DiHETrE**	0.709	0.385	0.658	0.297	0.686	0.170
**11-dh-TXB2**	149.939	114.425	115.509	137.988	84.521	60.813
**11-HDoHE**	1.911	1.267	0.966	1.291	0.426	0.547
**11-HETE**	0.856	0.441	0.929	1.260	0.560	0.289
**12(S)-HETE**	6.194	5.530	3.996	5.189	2.914	3.768
**12,13 EpODE**	106.504	60.093	83.735	63.376	21.257	61.587
**12,13-DiHOME**	4.883	5.224	3.160	2.418	2.961	3.608
**12,13-EpOME**	33.594	26.323	37.768	26.123	32.514	27.666
**12-HEPE**	1.796	1.539	0.978	1.514	0.447	0.636
**13(s)-HODE**	12.916	7.899	9.881	6.012	9.451	11.895
**13-HDoHE**	0.448	0.643	0.356	0.819	0.072	0.100
**13-HOTrE**	266.249	100.499	139.662	74.983	38.029	102.893
**13-OxoODE**	18.434	26.718	6.517	7.646	11.256	31.202
**14,15-DiHETrE**	0.811	0.175	0.813	0.295	0.876	0.224
**14-HDoHE**	2.913	2.896	1.570	2.068	0.923	1.459
**15 HETrE**	0.731	0.813	0.621	1.041	0.517	0.759
**15(S)-HETE**	1.235	0.901	1.278	1.165	0.929	0.407
**16-HDoHE**	0.326	0.191	0.431	0.930	0.162	0.102
**16-HETE**	0.146	0.386	0.138	0.239	0.130	0.180
**17-HDoHE**	0.843	1.065	1.112	2.007	0.508	0.602
**17-HETE**	91.956	82.045	55.573	47.235	38.497	41.410
**18-HEPE**	0.000	0.000	0.180	0.506	0.050	0.135
**18-HETE**	0.185	0.236	0.331	0.631	0.340	0.178
**19,20 DiHDoPE**	1.684	1.085	1.511	0.736	1.503	0.542
**20-HDoHE**	0.441	0.633	0.483	0.831	0.241	0.388
**4-HDoHE**	0.871	0.858	0.713	1.246	0.286	0.227
**5(S)-HETE**	1.818	1.109	1.592	1.871	0.947	0.454
**5,6-DiHETrE**	0.305	0.247	0.277	0.211	0.253	0.111
**5-HEPE**	0.675	0.750	0.839	1.405	0.282	0.398
**6-k-PGF1a**	0.640	0.300	0.734	0.246	0.695	0.217
**7-HDoHE**	0.178	0.274	0.338	0.905	0.087	0.132
**8,9-DiHETrE**	0.122	0.110	0.215	0.216	0.157	0.091
**8-HETE**	1.014	1.418	0.845	1.099	0.458	0.329
**9(S)-HODE**	11.120	8.512	8.607	6.278	8.317	10.030
**9,10,13 TriHOME**	22.668	52.820	3.186	7.370	4.607	15.732
**9,10-DiHOME**	4.283	3.583	3.656	3.932	3.163	3.657
**9,12,13 TriHOME**	11.948	26.975	2.072	3.288	2.691	8.509

**9-HOTrE**	0.935	0.703	0.815	0.764	0.680	0.510
**PGD2**	44.635	109.143	7.935	12.841	54.318	53.696
**PGE2**	0.264	0.187	0.360	0.175	0.341	0.138
**PGF2a**	0.141	0.373	0.325	0.509	0.217	0.347
**ResolvinD2**	6.331	11.296	6.726	10.624	4.861	2.986
**TXB2**	0.072	0.103	0.137	0.317	0.141	0.334

Supplemental Table S2 – Individual Plasma OxPCs by Smoking Status (ng/100µL)

OxPC	Never Smoked		Quit Smoking		Currently Smoking	
	Mean	SD	Mean	SD	Mean	SD
4-oxo-butyryl-PPC	0.351	0.259	0.185	0.217	0.154	0.123
POVPC	0.765	0.604	0.514	0.584	0.493	0.518
Succinoyl-PPC	1.233	0.516	0.868	0.797	0.645	0.299
PGPC	0.411	0.258	0.275	0.289	0.241	0.165
SOVPC	1.868	1.206	1.462	1.738	1.459	1.650
Furylbutanoyl-PPC	0.147	0.138	0.094	0.089	0.090	0.073
KOHA-PC	0.497	0.433	0.409	0.497	0.303	0.268
8octanoyl-PPC	1.934	1.160	1.421	1.721	1.399	0.978
SGPC	1.182	0.622	0.860	0.600	0.892	0.523
Acetal-POVPC	0.064	0.034	0.046	0.032	0.048	0.027
KOOA-PPC	0.413	0.203	0.271	0.242	0.211	0.184
PONPC	11.845	7.622	9.056	10.851	8.126	5.289
Furylbutanoyl-SPC	0.387	0.463	0.234	0.258	0.206	0.164
KOdiA-PPC	2.449	1.562	1.983	1.691	2.159	1.432
PAzPC	2.473	1.206	1.889	1.750	1.833	1.331
KOOA-SPC	3.303	4.055	4.279	8.765	2.965	4.966
SONPC	7.541	5.334	5.896	6.493	6.027	4.221
Furyloctanoyl-PPC	0.460	0.480	0.396	0.380	0.453	0.330
KOdiA-SPC	0.754	0.585	0.759	0.651	0.807	0.540
SAzPC	1.884	1.190	1.580	1.256	1.609	0.998
Acetal-PONPC	0.230	0.165	0.190	0.148	0.199	0.115
KODA-PPC	1.488	3.056	2.246	2.603	3.479	2.485
HODA-PPC	1.181	1.335	0.909	0.994	1.309	1.078
12-oxo-8,10-dodecendienoyl-PPC	0.055	0.047	0.048	0.033	0.035	0.025
Furyloctanoyl-SPC	0.783	0.756	0.994	1.462	0.848	0.716
KDdiA-PPC	1.485	1.218	1.565	1.028	1.884	0.988
HDdiA-PPC	0.060	0.067	0.059	0.160	0.026	0.014
Acetal-SONPC	0.132	0.044	0.090	0.063	0.079	0.040
10-OH-5,8,11-tridecatrienoyl-PPC	0.045	0.024	0.060	0.065	0.057	0.042
HODA-SPC	17.850	30.811	9.658	14.937	9.861	15.978
KDiA-SPC	0.758	0.361	1.067	1.532	0.839	0.683
HDiA-SPC	0.268	0.171	0.153	0.140	0.134	0.063
10-OH-5,8,11-tridecatrienoyl-SPC	45.793	38.441	71.144	125.482	46.778	19.342
PLPC-keto	1.592	1.251	1.479	1.003	1.575	0.811
PLPC-OH	1.905	1.040	1.677	0.808	1.601	0.440
PLPC-epoxy,keto	10.012	7.504	11.788	7.630	13.278	6.002
PLPC-OOH	1.532	0.976	1.609	0.913	1.774	0.753
15-deoxy-Δ12,14-isoPGJ2-PPC	0.261	0.209	0.203	0.173	0.207	0.195
PAPC-keto	0.758	0.511	0.680	0.395	0.700	0.341

PAPC-OHPAPC-epoxy	0.978	0.533	0.960	0.435	0.931	0.370
SLPC-keto	1.475	1.164	0.866	0.412	0.860	0.335
SLPC-OH	2.100	2.236	1.653	0.939	1.696	0.685
PLPC-OOH,keto	2.299	1.645	1.859	1.130	1.816	0.901
PLPC-OOH,OH	1.736	2.103	1.822	1.741	1.612	1.704
PLPC-diOH,epoxy	1.893	1.808	1.579	1.271	1.648	1.135
isoPG(A2,J2)-PPC	10.099	10.333	9.597	8.759	9.799	5.761
PAPC-OOH,	5.548	4.923	4.760	2.722	4.940	2.131
SLPC-epoxy,keto	2.254	1.820	2.579	1.378	2.732	1.122
SLPC-OOH	0.362	0.224	0.644	1.486	0.307	0.113
2,3-dinor-isoTxB2-PPC	0.216	0.154	0.128	0.093	0.132	0.073
15-deoxy-?12,14-isoPGJ2-PPC	0.378	0.278	0.342	0.501	0.264	0.102
SAPC-keto	0.556	0.397	0.546	0.359	0.591	0.308
SAPC-OH	0.530	0.265	0.473	0.220	0.426	0.147
PEIPC	0.710	0.534	0.579	0.304	0.585	0.222
isoPG(E2,I2,D2)-PPC	1.214	0.836	1.248	0.897	1.123	0.514
isoPGF2α-PPC	8.182	16.789	4.734	9.799	2.094	4.850
SLPC-OOH,OH	26.697	61.652	19.947	48.973	8.218	27.304
SLPC-triOH	0.360	0.222	1.540	7.317	0.272	0.122
SECPC	0.305	0.197	0.327	0.720	0.250	0.108
isoPG(A2,J2)-SPC	0.267	0.144	0.206	0.131	0.196	0.074
SAPC-OOH	0.280	0.109	0.255	0.101	0.239	0.058
PAPC-OOH,OH,keto	0.000	0.122	0.130	0.075	0.135	0.053
PAPC-diOOH	0.150	0.091	0.102	0.066	0.101	0.042
iso-TxB2-PPC	0.232	0.149	0.144	0.081	0.144	0.050
SLPC-diOOH,epoxy	0.462	0.339	0.409	0.283	0.442	0.241
SLPC-OOH,OH,keto	0.423	0.299	0.361	0.233	0.369	0.156
SEIPC	0.302	0.233	0.294	0.210	0.276	0.151
isoPG(E2,I2,D2)-SPC	0.170	0.139	0.165	0.170	0.154	0.073
isoPGF2α-SPC	0.154	0.085	0.131	0.115	0.131	0.062
PAPC-diOOH,OH	0.085	0.058	0.062	0.041	0.064	0.031
SLPC-diOOH,keto,epoxy	0.129	0.050	0.091	0.062	0.073	0.025
SLPC-diOOH,OH,epoxy	0.171	0.083	0.136	0.088	0.117	0.033
SAPC-OOH,diketo	0.180	0.115	0.159	0.095	0.143	0.039
SAPC-OOH,OH,keto	0.108	0.071	0.090	0.054	0.083	0.031
SAPC-diOOH	0.121	0.075	0.099	0.055	0.099	0.037
Iso-TxB2-SPC	0.261	0.188	0.259	0.189	0.284	0.145
PAPC-triOOH	0.555	0.389	0.443	0.344	0.491	0.252
SLPC-triOOH	0.075	0.058	0.056	0.042	0.055	0.030
SAPC-OOH,OH,epoxy	0.088	0.035	0.067	0.040	0.066	0.025
SAPC-diOOH,OH	0.050	0.037	0.043	0.034	0.038	0.017
PAPC-triOOH,OH	0.064	0.021	0.070	0.036	0.069	0.021
SAPC-triOOH	0.069	0.050	0.055	0.029	0.054	0.025

SAPC-triOOH,OH	0.044	0.015	0.053	0.063	0.043	0.036
